# Clonidine as an Additive to Local Anesthetics in Caudal Block for Postoperative Analgesia in Pediatric Surgery: A Systematic Review and Meta-Analysis

**DOI:** 10.3389/fmed.2021.723191

**Published:** 2021-09-14

**Authors:** Ye Wang, Qianqian Guo, Qi An, Lin Zhao, Meng Wu, Zhenggang Guo, Changsheng Zhang

**Affiliations:** ^1^Department of Anesthesiology, Peking University Shougang Hospital, Beijing, China; ^2^Anesthesia and Operation Centre, First Medical Centre of Chinese PLA General Hospital, Beijing, China

**Keywords:** anesthesia, caudal, epidural, clonidine, children, meta-analysis

## Abstract

**Background:** Clonidine is an anesthetic with favorable efficacy and safety profiles for caudal epidural block, but comparisons with other adjuvants need to be confirmed in pediatric patients.

**Aim:** To investigate the effects of clonidine as an adjuvant in caudal epidural block to improve the intraoperative and postoperative analgesia in pediatric surgery.

**Methods:** PubMed, Embase, and the Cochrane Library were searched for available papers published up to February 2021. The outcomes were pain score, duration of analgesia, complications, and number of analgesic requirements. The meta-analysis was performed using random-effects models.

**Results:** Fifteen randomized controlled trials (RCTs) were included. There were no differences between clonidine and the control drug regarding the duration of analgesia (SMD = −0.71, 95%CI: −1.64, 0.23; *I*^2^ = 95.5%, P_heterogeneity_ < 0.001), pain score (SMD = 0.35, 95%CI: −0.28, 0.98; *I*^2^ = 80.8%, P_heterogeneity_ < 0.001), and requirement for additional analgesia (OR = 8.77, 95%CI: 0.70, 110.58, *I*^2^ = 81.9%, P_heterogeneity_ = 0.004), but using clonidine resulted in fewer complications than the control drugs (OR = 0.33, 95%CI: 0.20, 0.54, *I*^2^ = 21.8%, P_heterogeneity_ = 0.217). The sensitivity analysis showed that the results were robust. A publication bias was observed.

**Conclusion:** Clonidine has the same efficacy as the other adjuvants for caudal epidural block for pediatric surgery but fewer complications. These results support clonidine as an adjuvant to local anesthetic, but additional studies should be conducted.

## Introduction

Caudal epidural block is widely popular for procedures below the umbilicus since it is a simple, safe, and reliable technique in pediatric patients (1, 2). Using landmark techniques and blind insertion, the success rate is >96% in pediatric patients (3, 4). The high reliability and ease of performance make caudal block one of the most suitable blocks in pediatric surgical patients. The commonly used local anesthetics for caudal block include bupivacaine, levobupivacaine, and ropivacaine. Still, their duration of action is short, and there are concerns of infection over their repeated use or continuous infusion (5). Therefore, adjuvant drugs are necessary to optimize the action of the local anesthetics (6). Various drugs such as opioids, dexmedetomidine, epinephrine, midazolam, ketamine, and neostigmine have been used as adjuvants for caudal epidural block but with various advantages, disadvantages, and adverse effects (7–10).

Clonidine is also used for single-injection caudal blocks (7). It is an α2-adrenergic agonist that produces analgesia without causing significant respiratory depression after caudal administration in children (11–13), although its use in children <3 months is debated because of a hypothetic risk of apnea (12, 13). The use of clonidine as an adjuvant for caudal block achieves appropriate analgesia but with the advantages of prolonged analgesia, reduced residual motor blockade, and increased margin of safety (14–16). A previous meta-analysis of only four trials showed that clonidine is as effective as morphine and with a more beneficial adverse effect profile in children (17), but it did not assess other anesthetics as controls and mainly focused on the side effects. A study compared clonidine vs. dexmedetomidine and showed that adjuvant dexmedetomidine was better than clonidine in terms of sedation, analgesia, and side effects (18), but El-Hennawy et al. (19) reported no differences between the two drugs in pediatric patients undergoing abdominal surgery, and Mota Bonisson et al. (20) reported no change in morphine consumption when adding clonidine to bupivacaine, but the sedation level was higher. Saini et al. (21) reported that clonidine was better than fentanyl as an adjuvant to ropivacaine for infraumbilical pediatric surgery. Evaluating the duration of analgesia and pain are also important factors in pediatric surgery. Given the conflicting results about the use of clonidine in such patients, additional analyses are necessary.

Therefore, this meta-analysis investigated the effects of clonidine as an adjuvant in caudal epidural block to improve the intraoperative and postoperative analgesia in pediatric surgery.

## Methods

### Literature Search

This systematic review and meta-analysis was performed according to the Preferred Reporting Items for Systematic Reviews and Meta-Analyses (PRISMA) guidelines (22). The research approach was designed using the PICOS principle (23). PubMed, Embase, and the Cochrane Library were searched for available papers published up to February 2021 using the MeSH terms “children,” “pediatric,” “bupivacaine,” “levobupivacaine,” “ropivacaine,” “clonidine,” and “analgesia,” as well as relevant key words, followed by screening based on the inclusion/exclusion criteria. The records were first evaluated based on the titles, followed by an assessment based on the abstracts and full-text. In the case of multiple using the same study population, only the most recent one matching the eligibility criteria was included.

### Eligibility Criteria

The eligibility criteria were (1) population: children, (2) local anesthetics: bupivacaine, ropivacaine, or levobupivacaine, (3) adjuvant in the intervention group: clonidine, (4) adjuvant in the control group: any drug other than clonidine, but not a placebo, (5) outcome: pain score, duration of analgesia, complications, and additional analgesia requirements, (6) study design: randomized controlled trials (RCTs), and (7) full-text article published in English. Reviews, meta-analyses, case reports, letters to the editor, and comments were excluded.

### Data Extraction

Study characteristics (authors, year of publication, country, and study design), patient characteristics (sex, sample size, weight, and previous surgery), anesthesia characteristics (local anesthetic, analgesia in control group, analgesic concentration, and usage), outcomes (duration of analgesia, pain score, need for additional analgesia, and complications were extracted by two different investigators Qi An and Lin Zhao) according to a pre-specified protocol. In multiple arm studies (6, 24), the sample size was divided by the times it has been compared, and the generated sample size was used as the sample size of each subgroup, as previously described (25). Discrepancies were solved by discussion until a consensus was reached.

### Pain Evaluation

The pain was evaluated using the Objective Pain Score (OPS) (24, 26–29), Children's Hospital of Eastern Ontario Pain Scale (CHEOPS) (30, 31), Face, Legs, Activity, Cry, and Consolability (FLACC) (6, 9, 19, 32–34), Children and Infants Postoperative Pain Scale (CHIPPS) (35), pinprick at each dermatome (36), or a visual analog scale (30). When possible, the pain was evaluated as a continuous variable for comparisons between the two groups. The studies that reported pain as a categorical variable were analyzed separately.

### Quality of the Evidence

The level of evidence of all articles was assessed independently by two authors (YeWang and QianqianGuo) according to Version 2 of the Cochrane risk-of-bias assessment tool for randomized trials (RoB 2) (37, 38). The studies were evaluated using Grading of Recommendations Assessment Development and Evaluation (GRADE) (39). Discrepancies in the assessment were resolved through discussion until a consensus was reached.

### Statistical Analysis

All analyses were performed using STATA SE 14.0 (StataCorp, College Station, Texas, USA). The standardized mean difference (SMD) and 95% confidence intervals (CI) were used for continuous variables, and odds ratio (OR) with 95%CI were used for categorical variables. Statistical heterogeneity among studies was calculated using Cochran's *Q*-test and the I^2^ index. An *I*^2^ > 50% and *Q*-test *P* < 0.10 indicated high heterogeneity. The meta-analysis was performed using random-effects models. *P*-values < 0.05 were considered statistically significant. Sensitivity analyses were performed to assess the robustness of the original analyses. In addition, subgroup analyses were performed. Finally, potential publication bias was assessed using Egger's test, Begg's test, and the trim-and-fill method (37).

## Results

### Selection of the Studies

**Figure 1:**
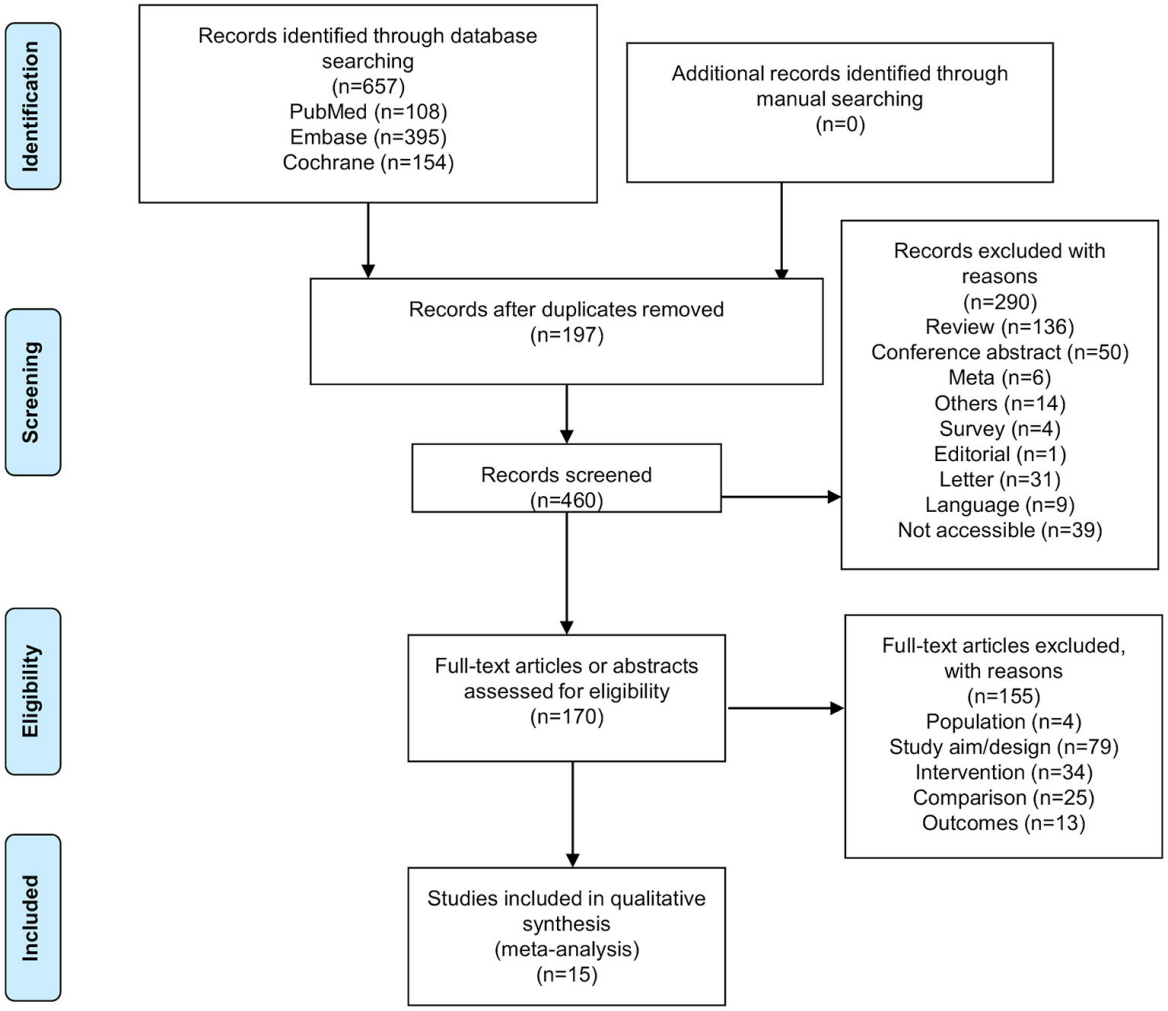
Flow diagram of the study selection process.

Figure 1 presents the study selection process. The initial database search identified 657 records. After removing the duplicates, 460 records were screened, and 290 were excluded. Then, 170 abstracts or full-text articles were assessed for eligibility, and 155 were excluded (population, *n* = 4; study aim/design, *n* = 79; intervention, *n* = 34; comparison, *n* = 25; outcomes, *n* = 13). Finally, 15 articles were included.

Table 1 presents the characteristics of the studies and patients. Fifteen studies (17 datasets; 770 patients) were included. The control groups included ketamine (24, 26, 28, 31), tramadol (27, 35), fentanyl (24, 30, 36, 40), dexmedetomidine (19, 34), morphine (6, 29, 33), midazolam (9), and hydromorphone (6). The local anesthetics included ropivacaine (6, 26, 31, 40), bupivacaine (9, 19, 24, 27–30, 33, 34, 36), and levobupivacaine (35). Supplementary Table 1 shows the quality evaluation. Seven studies had a low risk of bias, while eight studies had an unclear risk of bias for at least one item of the RoB 2 tool. Supplementary Table 2 shows the GRADE analysis. The pain score and the duration of analgesia had critical importance, and both showed moderate certainty. The requirement for additional analgesia was important and showed a high level of certainty. Complications were important and displayed a moderate level of certainty.

**Table 1:** Literature search and characteristics of the included studies.

**References**	**Design**	**Country**	**Surgery**	**Control**	**Local anesthetics**	**Sample size**		**Age (year, mean, or median)**		**Weight, kg**		**Analgesic concentration and usage**		
						**Clonidine**	**Control**	**Clonidine**	**Control**	**Clonidine**	**Control**	**Clondine**	**Bupi/ropi/ levobupi**	
Akbas et al. (26)	RCT	Turkey	Inguinal hernia repair and circumcision	Ketamine	Ropivacaine	25	25	6.08 (2.87)	5.92 (3.14)	20.34 (8.27)	20.36 (7.8)	1 μg/kg	0.2%, 0.75 ml/kg	lower
Amitha et al. (27)	RCT	India	Lower abdominal/lower limb surgery	Tramadol	Bupivacaine	30	30	8.26 (2.98)	9.03 (2.94)	22.16 (7.78)	26.76 (6.74)	2 μg/kg	0.25%, 0.5 ml/kg	lower
Constant et al. (30)	RCT	France	Bilateral correction of vesicoureteral reflux	Fentanyl	Bupivacaine	16	15	3.6 (0.5–9)	3.8 (1.8–6.5)	15 (5)	16 (4)	1.5 μg/kg	0.25%	lower
Cook et al. (28)	RCT	UK	Unilateral orchidopecy	Ketamine	Bupivacaine	20	20	5.02 (1.3–9)	6.03 (1.5–9)	20.1 (8.8)	23.1 (7.1)	2 μg/kg	0.25%. 1 mL/kg	lower
De Negri et al. (31)	RCT	Italy	Hernia repair/orchidopexy	S-ketamine	Ropivacaine	20	19	3 (1.5)	2.7 (1.2)	12 (7)	13 (5)	2 μg/kg	0.2%, 2 mg/kg	lower
El-Hennawy et al. (19)	RCT	Egypt	Lower abdominal surgery	Dexmedetomidine	Bupivacaine	20	20	3.8 (0.5–5.8)	3.3 (0.7–5)	16 (4.9)	14 (5.2)	2 μg/kg	0.25%, 1 ml/kg	lower
Fernandes et al. (33)	RCT	Brazil	Infraumbilical urological and genital procedures	Morphine	Bupivacaine	20	20	4.7 (2.7)	4.8 (2.6)	17.9 (7.4)	21.6 (11.2)	1 μg/kg	0.166%, 1.0 ml/kg	lower
Luz et al. (29)	RCT	Australia	Orchidopexy, hernia repair, circumcision	Morphine	Bupivacaine	18	18	2.8 (0.6–6)	2.7 (0.7–6.3)	13.9 (7.2–20)	14.2 (7.6–25)	1 μg/kg	0.18%, 1.5 ml/kg	lower
Parag et al. (36)	RCT	India	Hernia repair	Fentanyl	Bupivacaine	40	40	5.4 (2.46)	5.8 (2.63)	16.58 (3.82)	17.7 (6.3)	1 μg/kg	0.5%,	lower
Rawat et al. (35)	RCT	India	Perineal surgery	Tramadol	Levobupivacaine	22	22	4.14 (1.05)	4.23 (2.02)	11.64 (2.25)	12.2 (2.6)	1 μg/kg	0.25%. 1 mg/kg	lower
Sanwatsarkar et al. (9)	RCT	India	Infraumbilical surgery	Midazolam	Bupivacaine	25	25	6.28 (1.21)	6.16 (1.11)	15.48 (3.34)	14.96 (2.88)	1 μg/kg	0.25%. 1 mg/kg	lower
Shukla et al. (40)	RCT	Etawah	Infraumblical	Fentanyl	Ropivacaine	45	45	5.1 (3–7)	4.1 (3.3–7.8)	18 (6.2)	15 (7.2)	2 μg/kg	0.25%, 1 ml/kg	
Singh et al. (24)	RCT	Nepal	Below umbilical surgeries	Fentanyl	Bupivacaine	10	20	5.45 (2.5)	5.7 (2.8)	14.7 (3.8)	14.75 (4)	1 μg/kg	0.25%, 0.75 ml/kg	lower
Singh et al. (24)	RCT	Nepal	Below umbilical surgeries	Ketamine	Bupivacaine	10	20	5.45 (2.5)	5.3 (1.8)	14.7 (3.8)	16.85 (4.19)	1 μg/kg	0.25%, 0.75 ml/kg	lower
Singh et al. (34)	RCT	India	Upper abdominal surgery	Dexmedetomidine	Bupivacaine	25	25	2.9 (1–6)	2.8 (1.5–6)	11.3 (3.1)	11.8 (2.18)	2 μg/kg	0.2%, 1.25 ml/kg	upper
Vetter et al. (6)	RCT	USA	Ureteral reimplantation	Morphine	Ropivacaine	10	20	3.5 (1.7)	3.4 (1.8)	16 (6)	15 (4)	2 μg/kg	0.2%, 1.0 ml/kg	lower
Vetter et al. (6)	RCT	USA	Ureteral reimplantation	Hydromorphone	Ropivacaine	10	20	3.5 (1.7)	3.4 (1.8)	16 (6)	16 (5)	2 μg/kg	0.2%, 1.0 ml/kg	lower

### Duration of Analgesia

**Figure 2:**
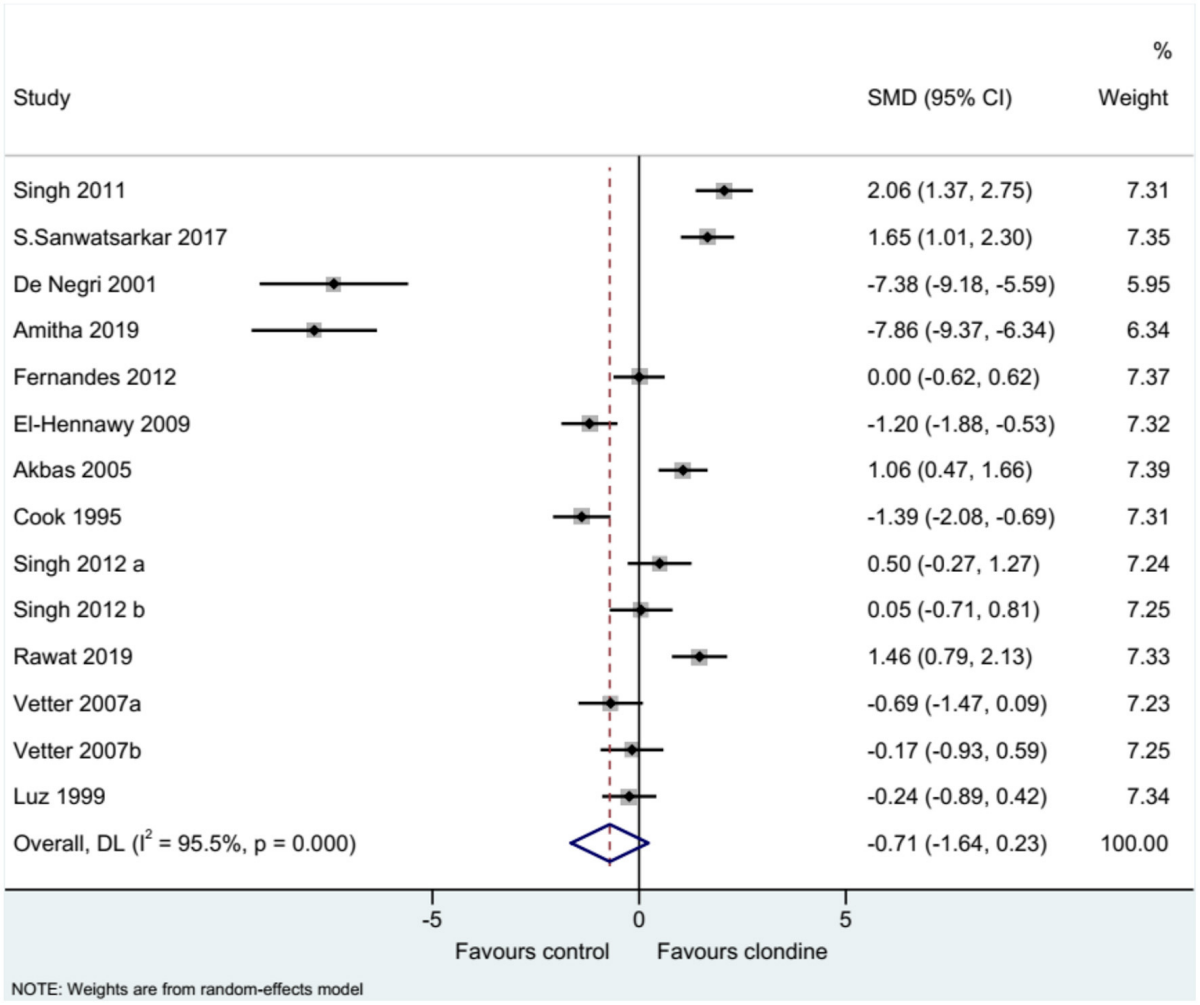
Duration of analgesia duration.

**Figure 3:**
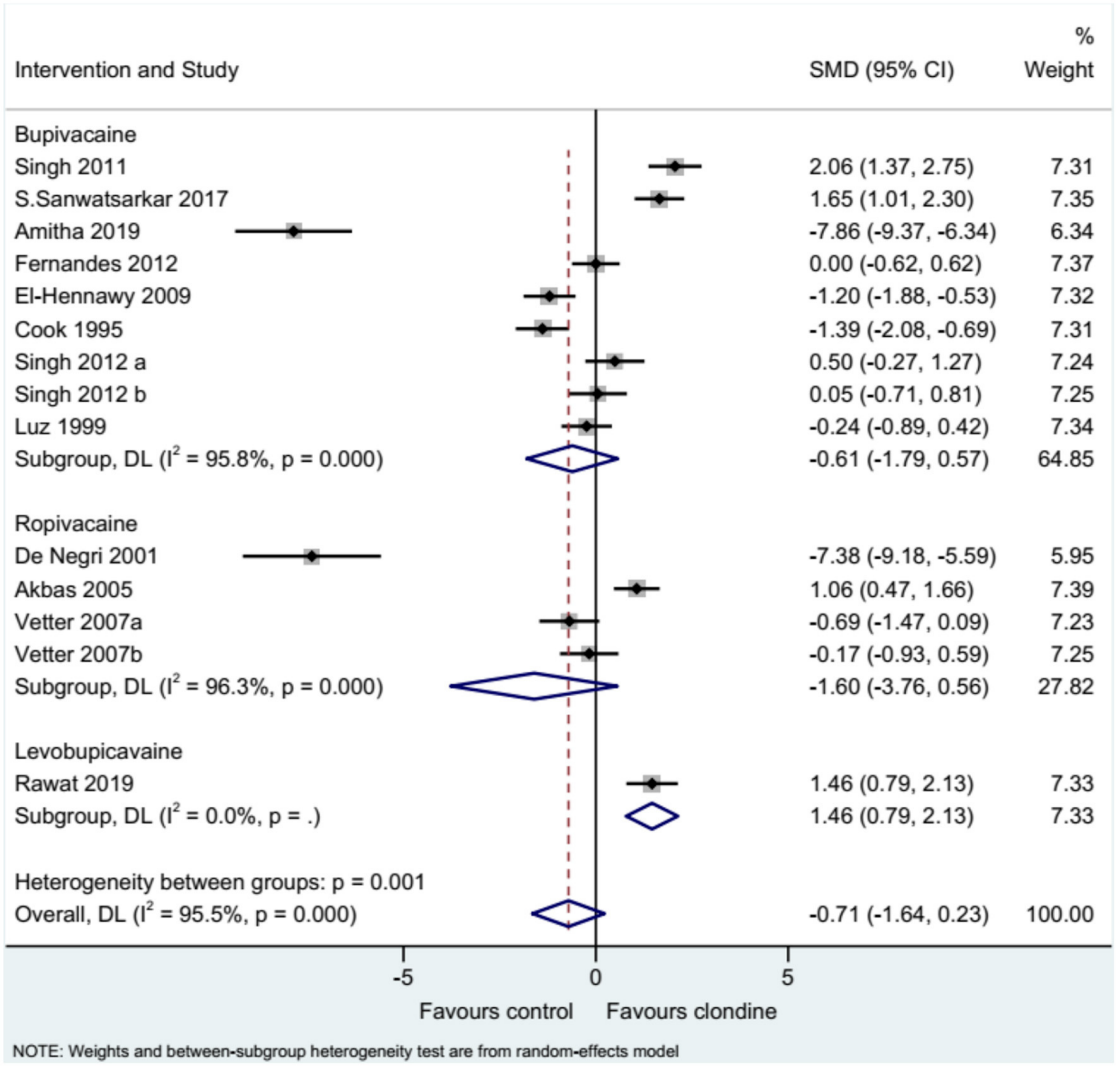
Subgroup analysis of analgesia duration by various local anesthetics.

**Figure 4:**
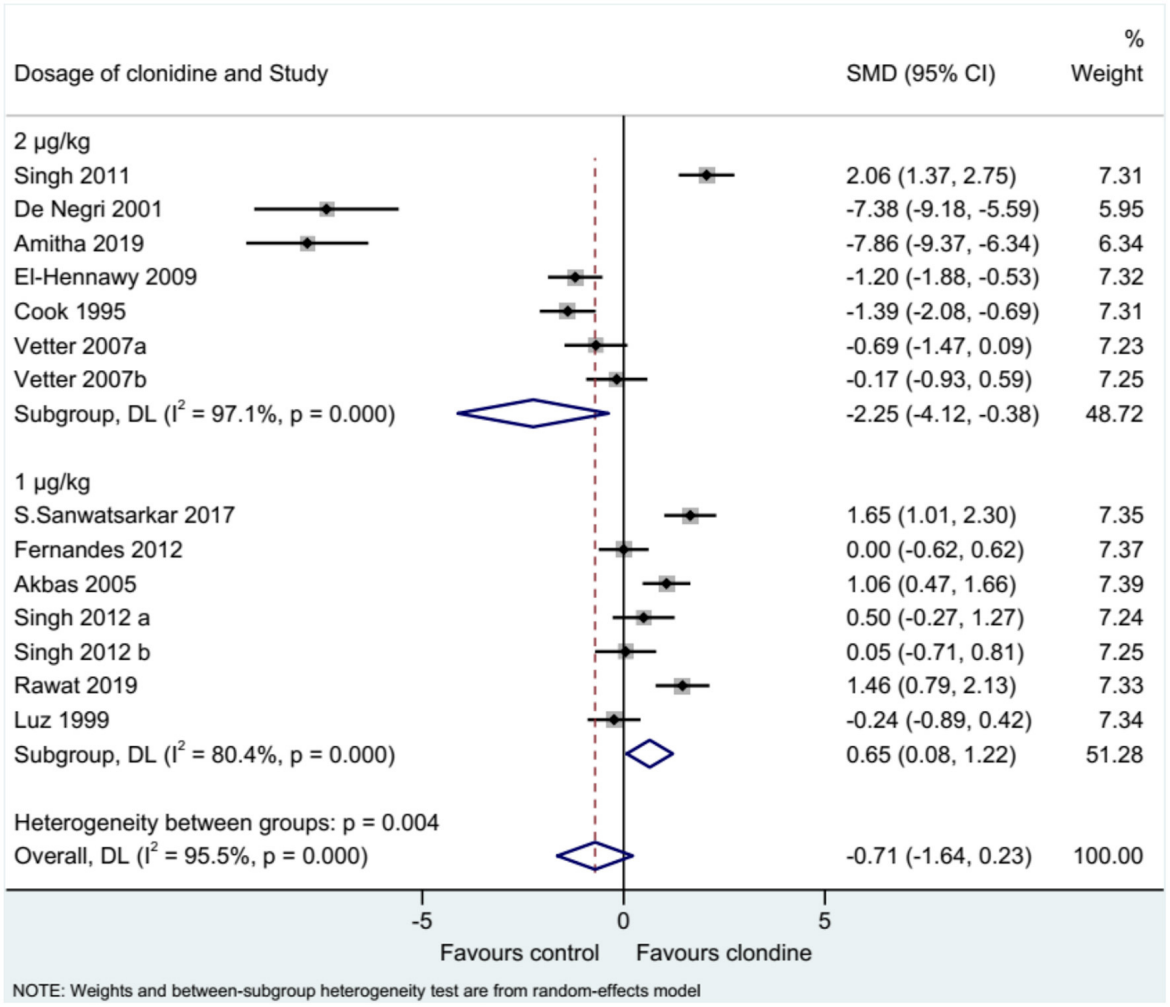
Subgroup analysis of analgesia duration by the dosage of clonidine.

Twelve studies (14 datasets) reported the duration of analgesia. There was no difference between clonidine and the control drug regarding the duration of analgesia (SMD = −0.71, 95%CI: −1.64, 0.23; *I*^2^ = 95.5%, P_heterogeneity_ < 0.001) (Figure 2). A subgroup analysis was performed according to the type of local anesthetic, and there were no differences between clonidine and the control drug in the presence of bupivacaine (SMD = −0.61, 95%CI: −1.79, 0.57, *I*^2^ = 95.8%, P_heterogeneity_ < 0.001) or ropivacaine (SMD = −1.60, 95%CI: −3.76, 0.56, *I*^2^ = 96.3%, P_heterogeneity_ < 0.001), but one study favored clonidine with levobupivacaine (SMD = −1.46, 95%CI: 0.79, 2.13) (Figure 3). Regarding the dose of clonidine, the use of clonidine 2 μg/kg favored the control drug (SMD = −2.25, 95%CI: −4.12, −0.38, *I*^2^ = 97.1%, P_heterogeneity_ < 0.001), while the use of clonidine 1 μg/kg favored clonidine (SMD = 0.65, 95%CI: −0.08, 1.22, *I*^2^ = 80.4%, P_heterogeneity_ = 0.004) (Figure 4).

### Pain Score

**Figure 5:**
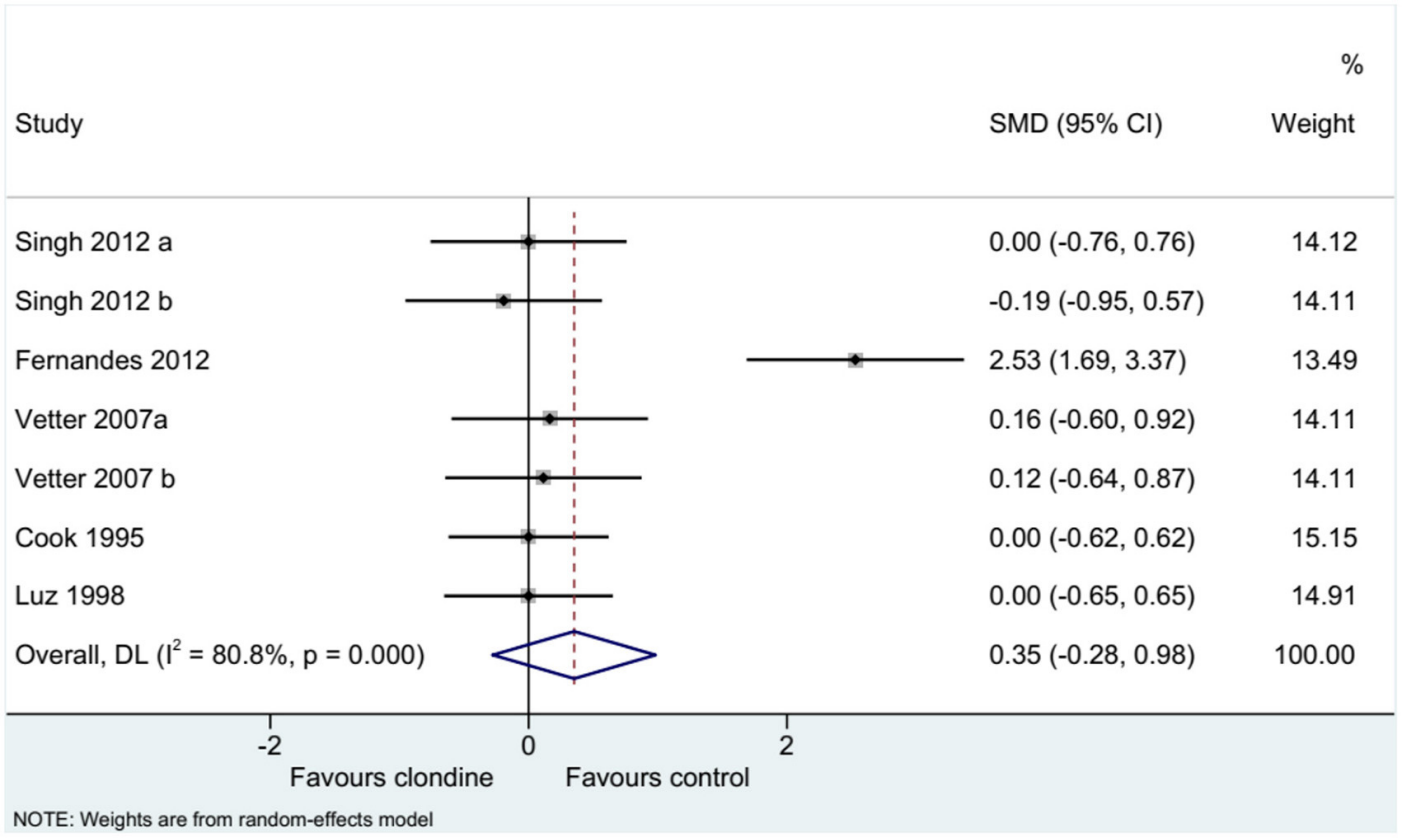
Pain score (continuous variables).

**Figure 6:**
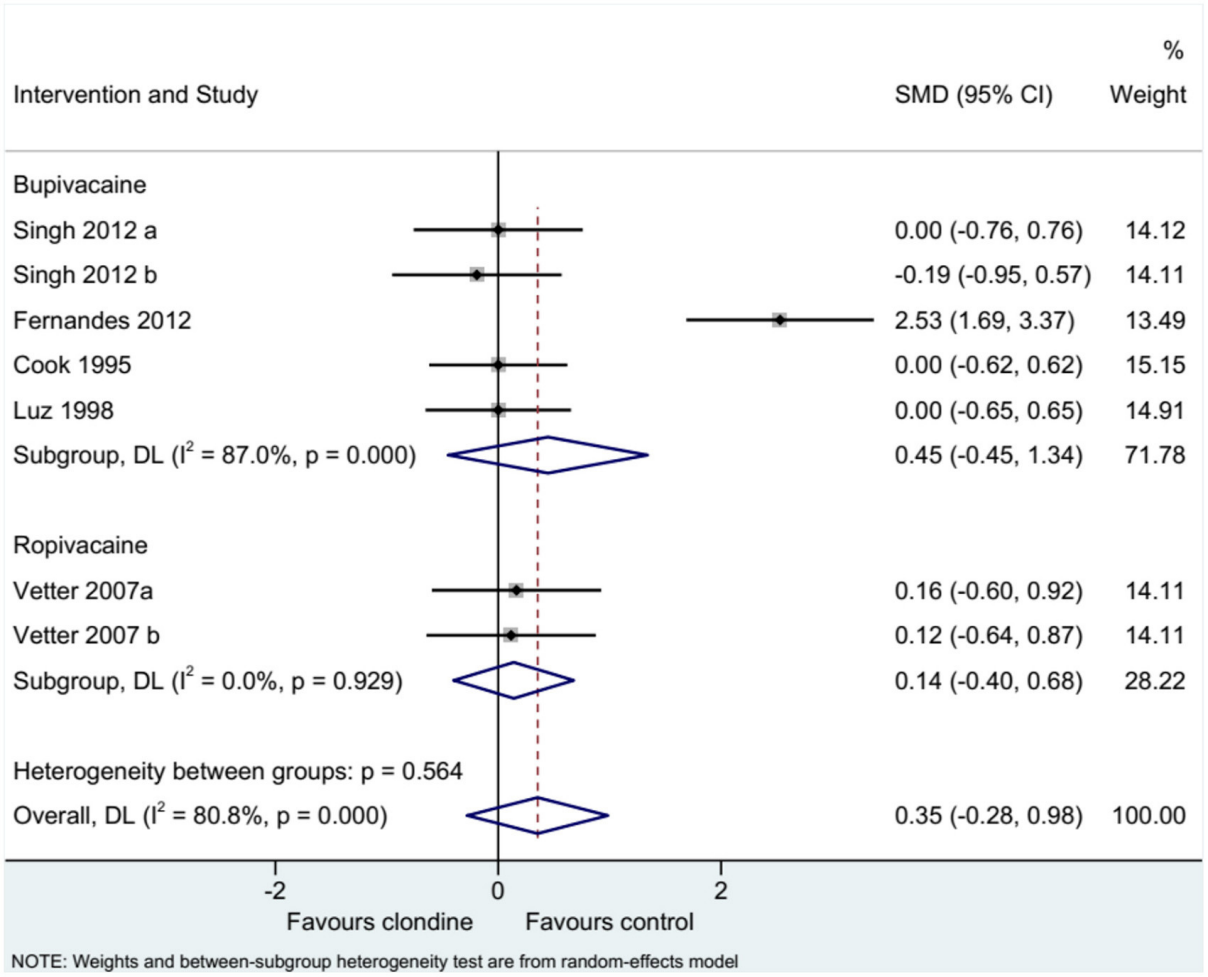
Subgroup analysis of pain score by local anesthetic.

**Figure 7:**
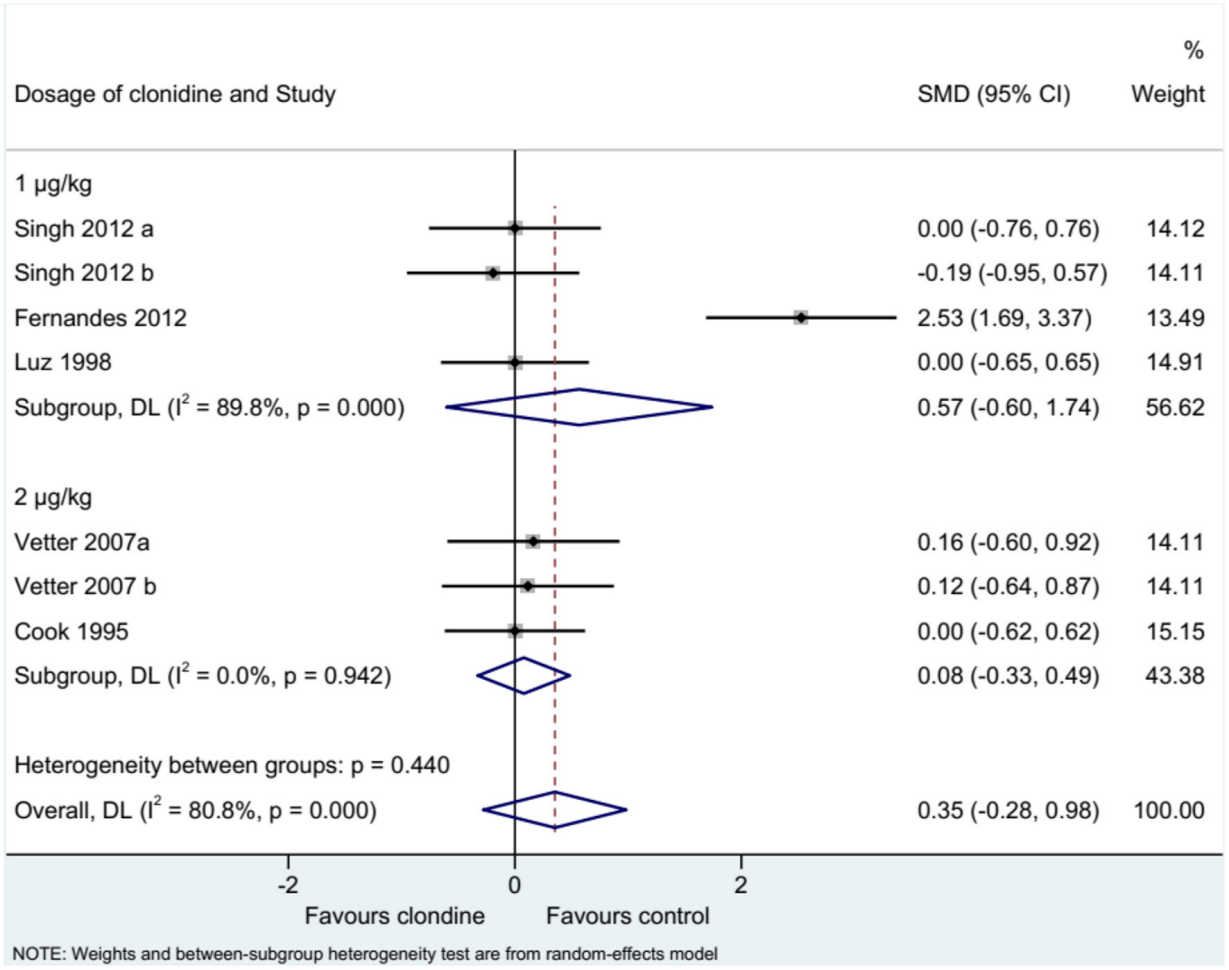
Subgroup analysis of pain score by the dosage of clonidine.

**Figure 8:**
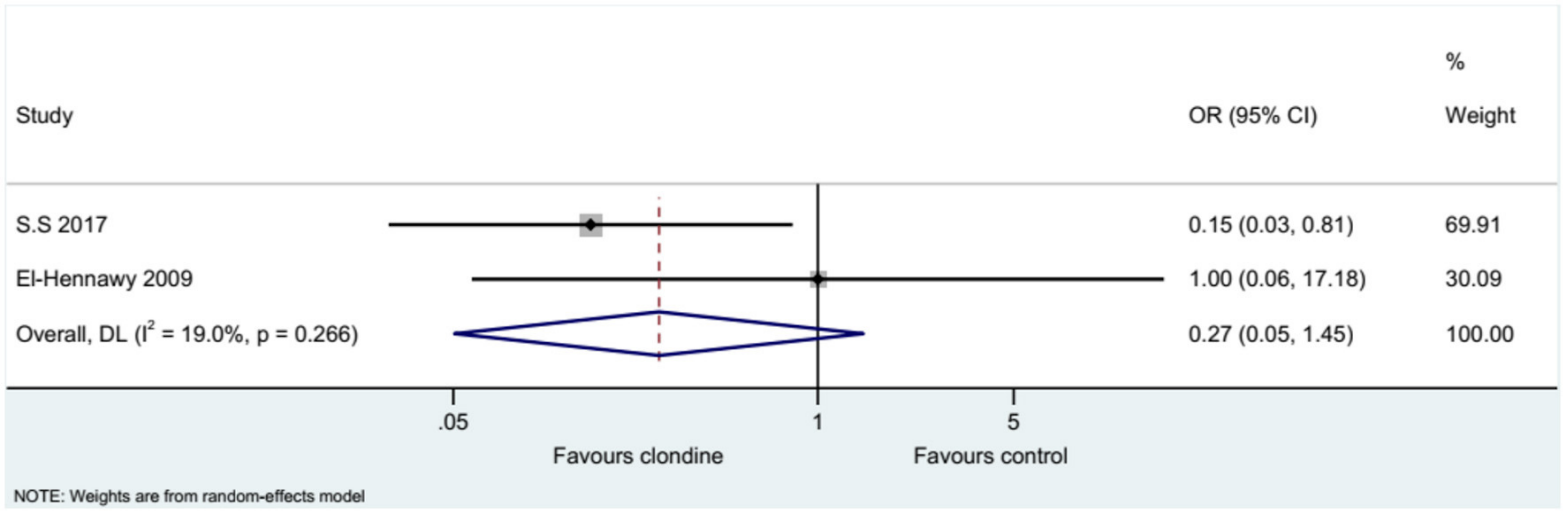
Pain score (categorical variables).

Five studies (seven datasets) analyzed pain (as a continuous variable). There were no differences between clonidine and the control drugs regarding pain (SMD = 0.35, 95%CI: −0.28, 0.98; *I*^2^ = 80.8%, P_heterogeneity_ < 0.001) (Figure 5). Similar results were obtained when considering buvicaine (SMD = 0.45, 95%CI: −0.45, 1.34, *I*^2^ = 87.0%, P_heterogeneity_ < 0.001) or ropivacaine (SMD = 0.14, 95%CI: 0.40, 0.68, *I*^2^ = 0.0%, P_heterogeneity_ = 0.929) as the local anesthetic (Figure 6), or when considering clonidine 2 μg/kg (SMD = 0.57, 95%CI: −0.60, 1.74, *I*^2^ = 89.8%, P_heterogeneity_ < 0.001) or 1 μg/kg (SMD = 0.08, 95%CI: −0.33, 0.49, *I*^2^ = 0.0%, P_heterogeneity_ = 0.440) (Figure 7). Two studies examined pain as a categorical variable showed no difference between clonidine and the control drugs (OR = 0.27, 95%CI: 0.05, 1.45, *I*^2^ = 19.0%, P_heterogeneity_ = 0.266) (Figure 8).

### Requirement for Additional Analgesia

**Figure 9:**
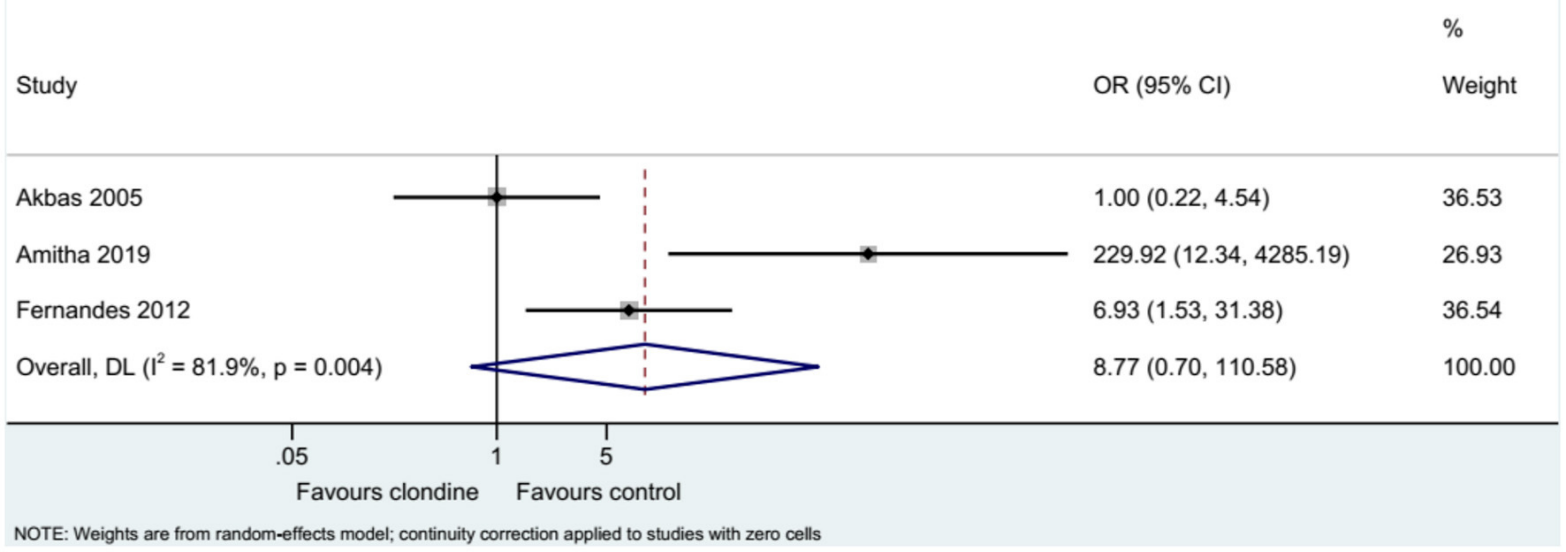
Total numbers of analgesia of post-requirements.

**Figure 10:**
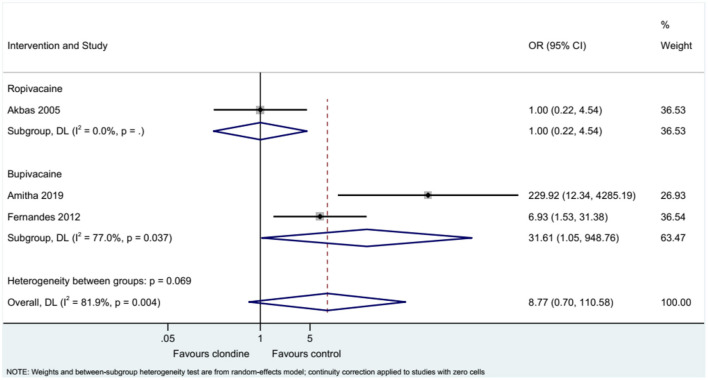
Subgroup analysis of post-requirements by local anesthetic.

**Figure 11:**
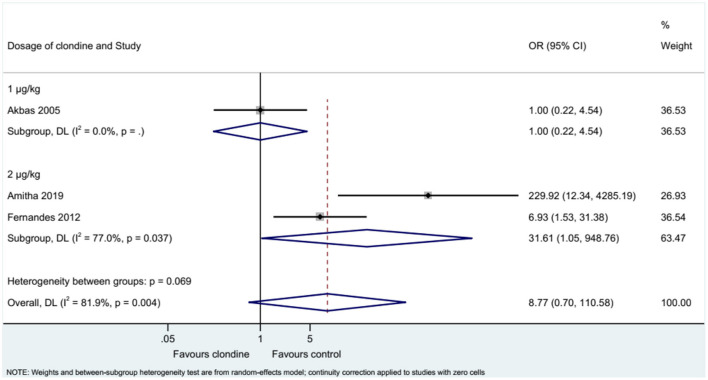
Subgroup analysis of post-requirements by the dosage of clonidine.

Three studies examined the requirement for analgesia and showed no difference between clonidine and the control drugs (OR = 8.77, 95%CI: 0.70, 110.58, *I*^2^ = 81.9%, P_heterogeneity_ = 0.004) (Figure 9). The requirement for analgesia was not influenced by ropivacaine (OR = 1.00, 95%CI: 0.22, 4.54), but using bupivacaine favored the control drugs in terms of the requirement for additional analgesia (OR = 31.61, 95%CI: 1.05, 948.76, *I*^2^ = 77.0%, *P*_heterogeneity_ = 0.037) (Figure 10). The requirement for analgesia was not influenced by clonidine 1 μg/kg (OR = 1.00, 95%CI: 0.22, 4.54), but using clonidine 2 μg/kg favored the control drugs in term of requirement for analgesia (OR = 31.61, 95%CI: 1.05, 948.76, *I*^2^ = 77.0%, P_heterogeneity_ = 0.037) (Figure 11).

### Complications

**Figure 12:**
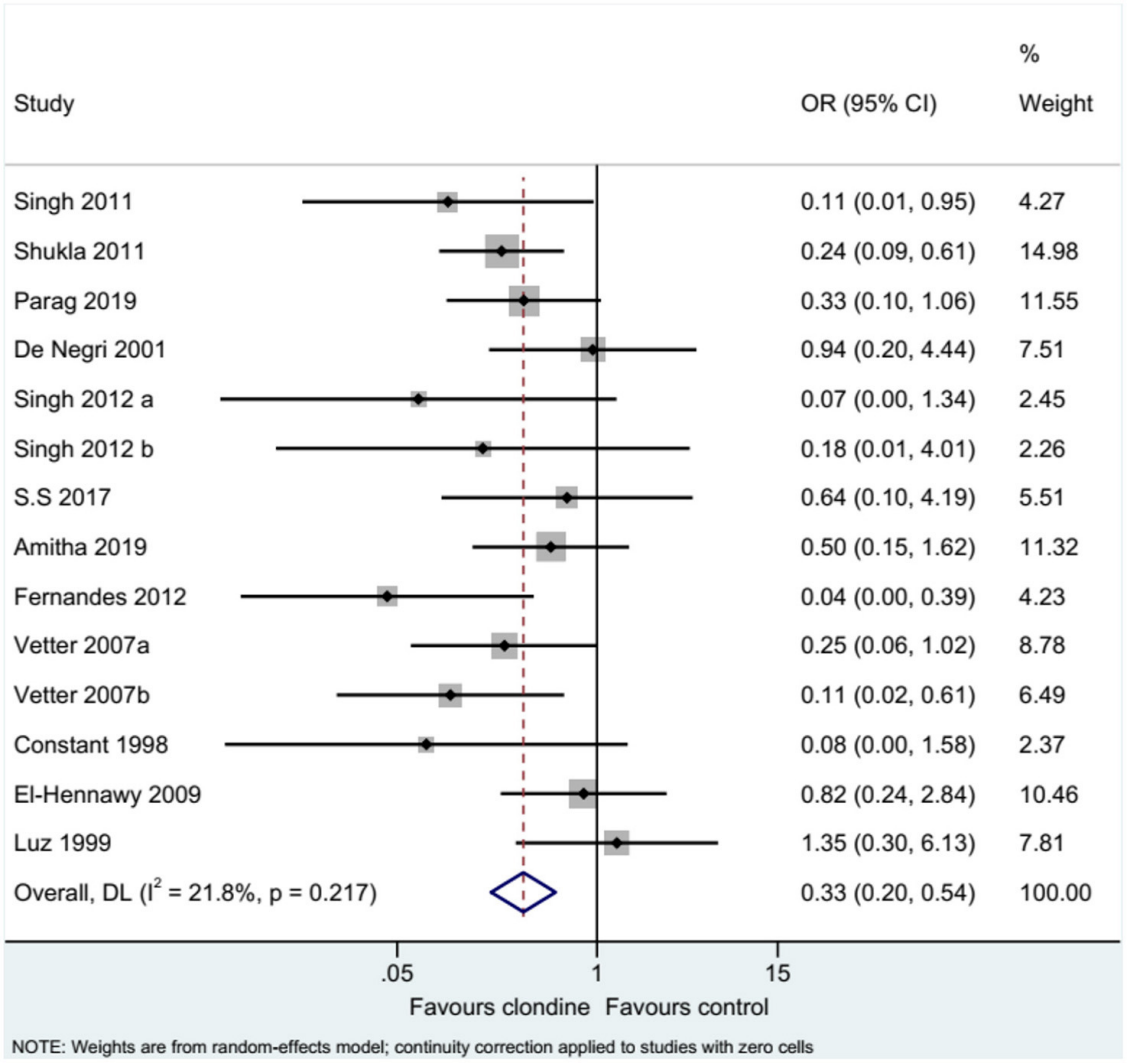
Complications.

**Figure 13:**
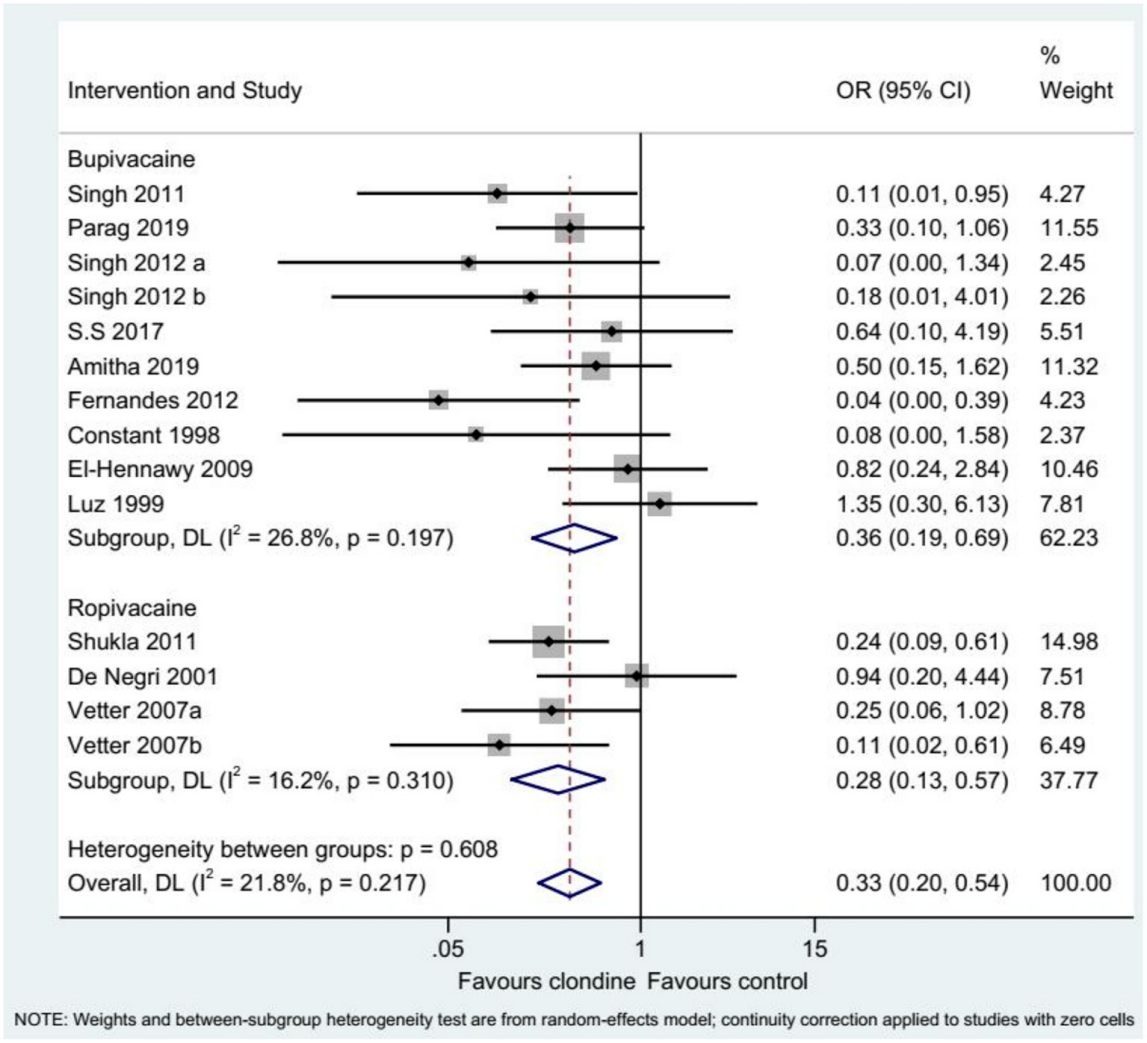
Subgroup analysis of complications by local anesthetic.

**Figure 14:**
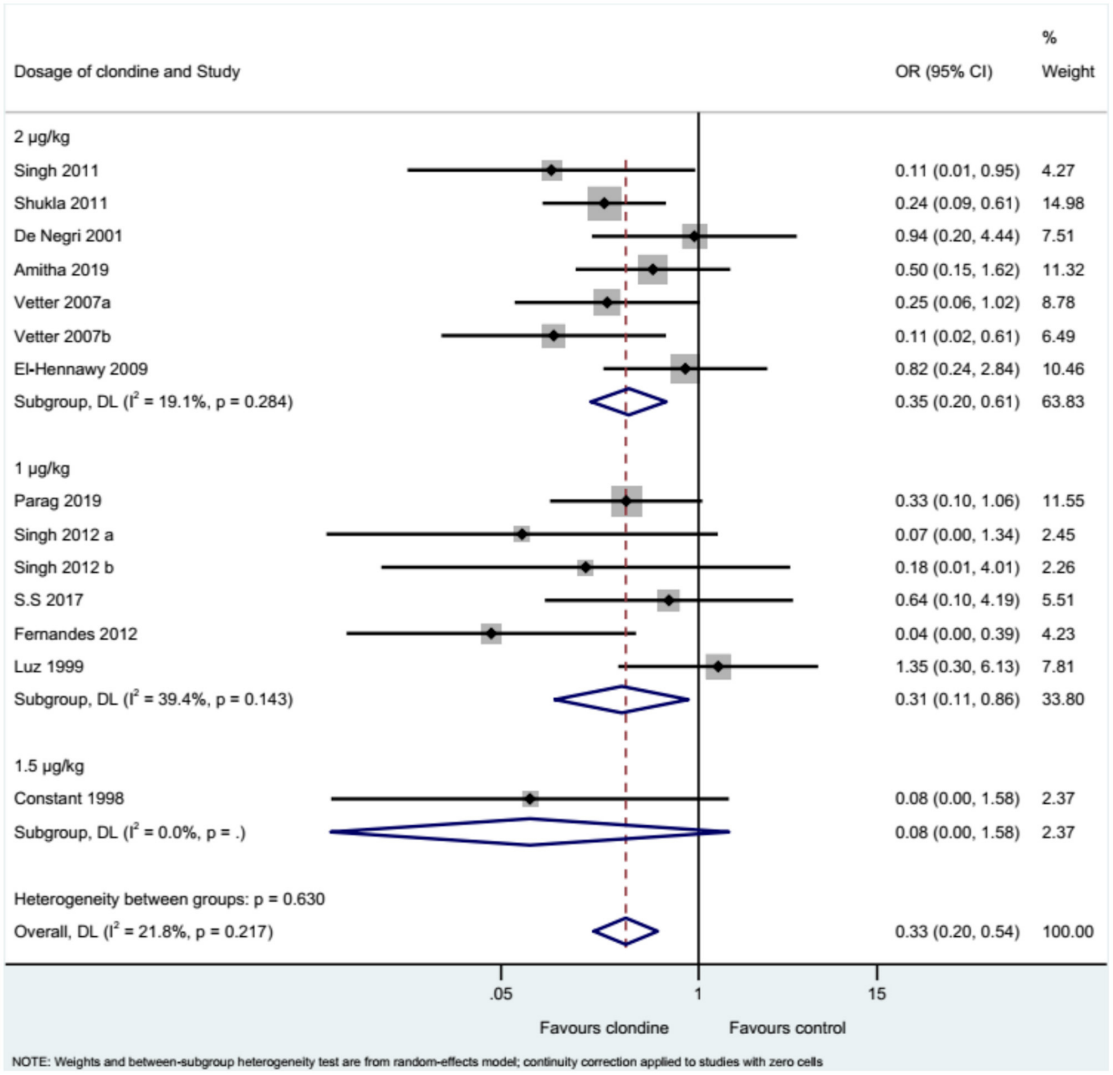
Subgroup analysis of complications by the dosage of clonidine.

Twelve studies (14 datasets) reported the complications of caudal epidural block. Using clonidine resulted in fewer complications than the control drugs (OR = 0.33, 95%CI: 0.20, 0.54, *I*^2^ = 21.8%, P_heterogeneity_ = 0.217) (Figure 12). Similar results were observed when using either bupivacaine (OR = 0.36, 95%CI: 0.19, 0.69, *I*^2^ = 26.8%, P_heterogeneity_ = 0.197) or ropivacaine (OR = 0.28, 95%CI: 0.13, 0.57, *I*^2^ = 16.2%, P_heterogeneity_ = 0.310) as the local anesthetic (Figure 13), or when using clonidine 2 μg/kg (OR = 0.35, 95%CI: 0.20, 0.61, *I*^2^ = 19.1%, P_heterogeneity_ = 0.284) or clonidine 1 μg/kg (OR = 0.31, 95%CI: 0.11, 0.86, *I*^2^ = 39.4%, P_heterogeneity_ = 0.143), but not clonidine 1.5 μg/kg (OR = 0.08, 95%CI: 0.00, 1.58) (Figure 14).

### Sensitivity Analysis

Supplementary Figures 1–3 show that the results of analgesia duration, the requirement for additional analgesia, and complications were robust.

### Publication Bias

Begg's test (*P* = 0.049) and Egger's test (*P* = 0.001) indicate the presence of a significant publication bias. The results of the trim-and-fill analysis suggest that an additional 14 RCTs would be necessary to change this conclusion (Supplementary Figure 4).

## Discussion

Clonidine is an anesthetic with favorable efficacy and safety profiles for use in caudal epidural block in children. This meta-analysis aimed to investigate the effects of clonidine as an adjuvant in caudal epidural block to improve the intraoperative and postoperative analgesia in pediatric surgery. The results suggest that clonidine has the same efficacy as the control drugs for caudal epidural block for pediatric surgery but fewer complications. Thus, these results support clonidine as an adjuvant to local anesthetic, but additional studies should be conducted.

A previous meta-analysis compared clonidine and morphine for caudal epidural block using only four studies and only morphine as control (17). Their results showed no differences regarding analgesia duration and the need for rescue analgesia, as in the present study and a meta-analysis of clonidine vs. Dexmedetomidine (18). Still, many drugs are available besides morphine for caudal block, limiting the generalizability of that previous meta-analysis. A review suggested that epidural clonidine might be more effective than opioids to manage chronic pain (41). A meta-analysis reported that dexmedetomidine had better analgesic effects than clonidine for hysterectomy (42). In the present meta-analysis, many studies reported no difference between clonidine and the comparator regarding analgesia duration (6, 24, 29, 33), while some studies favored either clonidine (9, 26, 34, 35) or the comparator (19, 27, 28, 31). Of course, the nature of the comparator might play an important role in the conclusions of the individual studies.

Clonidine inhibits the release of nociceptive neurotransmitters (33). The adverse effects of clonidine are mainly related to the excitation of α2 inhibitory neurons in the medulla vasomotor center, leading to decreased norepinephrine secretion (43). In addition, clonidine decreases the electrical activity of preganglionic parasympathetic nerves and reduces sympathetic drive, resulting in bradycardia (43, 44). Still, the other drugs commonly used for caudal epidural block also have adverse effects, like hemodynamic effects for dexmedetomidine (45), gastrointestinal dysmotility, nausea/vomiting, pruritus, and respiratory depression for opioids (12, 46), and neuronal apoptosis for ketamine (12, 46, 47). In the present study, the complications were less important with clonidine than with the other drugs. The meta-analysis by Goyal et al. (17) also reported less nausea/vomiting with clonidine than with morphine.

In the present meta-analysis, nearly all analyses showed significant heterogeneity. This heterogeneity could be explained by differences among the included studies regarding the age of the children, the type of surgeries, the comparator drug, the local anesthetic, and the dose of clonidine. Subgroup analyses were performed regarding the local anesthetics and the clonidine dose. The results showed that using bupivacaine instead of ropivacaine was associated with a higher requirement for additional analgesia than the control group, while the choice of local anesthetic did not influence the other parameters. Regarding the dose of clonidine, using a higher dose favored the control drugs in analgesia duration and requirement for additional analgesia while having no impact on pain and complications. Therefore, using a lower dose (1 μg/kg) could be conducive to better results, especially regarding the duration of analgesia. These results are still surprising because Lee et al. (48) reported longer analgesia with a higher dose. Still, Singh et al. (24) reported that a lower dose of clonidine combined with bupivacaine fared better than the other drug combinations. Therefore, the subgroup analyses in the present study must be taken with caution, especially considering the different combinations of drugs and clonidine doses. Additional studies are necessary on this point.

Assessment of pain is complex in children and can be based only on physiological and behavioral parameters since young children cannot communicate verbally (49). The exact source of pain is difficult to determine, but understanding the various patterns of cues used by children to manifest pain is a complex undertaking (49). Different tools are recommended according to the verbal/non-verbal status of the patients (50). In addition, the included studies used various pain scale assessments, including OPS (24, 26–29), CHEOPS (30, 31), FLACC (6, 9, 19, 32–34), CHIPPS (35), pinprick at each dermatome (36), or a VAS (30), participating in heterogeneity. Even if all these assessments assess pain, they use different indicators (51). CHEOPS is validated for children of 1–7 years, FLACC for 2 months-7 years, CHIPPS for 0–5 years, OPS for 8 months-13 years, and VAS starting from 5 years (51). In addition, Sanwatsarkar et al. (9) and El-Hennawy et al. (19) presented their pain results in categorical variables based on the FLACC pain scale.

The strengths of this meta-analysis include a relatively large number of studies (only RCTs, leading to a high level of evidence) and a large number of patients. Still, this meta-analysis has limitations. As for any such study, a meta-analysis inherits the limitations of all the included studies, and caution must be applied while extrapolating the results. Two studies included multiple arms (6, 24), which were dealt with using a specific method (25). Although this method might introduce bias, it is a feasible way to deal with the problem of multiple arm studies being compared repeatedly.

In conclusion, clonidine has the same efficacy as the other adjuvants for caudal epidural block for pediatric surgery but fewer complications. These results support clonidine as an adjuvant to local anesthetic, but additional studies should be conducted because of a significant publication bias.

## Data Availability Statement

The original contributions presented in the study are included in the article/Supplementary Material, further inquiries can be directed to the corresponding author/s.

## Author Contributions

YW conceived and coordinated the study, designed, performed and analyzed the experiments, and wrote the paper. QG, QA, LZ, MW, ZG, and CZ carried out the data collection and data analysis and revised the paper. All authors reviewed the results and approved the final version of the manuscript.

## Funding

This study was supported by Wu Jieping Medical Foundation (320.6750.19018), Peking University Shougang Hospital Scientific Research and Development Funds (2019-yuan-lc-10).

## Conflict of Interest

The authors declare that the research was conducted in the absence of any commercial or financial relationships that could be construed as a potential conflict of interest.

## Publisher's Note

All claims expressed in this article are solely those of the authors and do not necessarily represent those of their affiliated organizations, or those of the publisher, the editors and the reviewers. Any product that may be evaluated in this article, or claim that may be made by its manufacturer, is not guaranteed or endorsed by the publisher.
