# Supplementary material for: Clonidine as an Additive to Local Anesthetics in Caudal Block for Postoperative Analgesia in Pediatric Surgery: A Systematic Review and Meta-Analysis
Source: Front Med (Lausanne). 2021 Sep 14;8:723191. doi: 10.3389/fmed.2021.723191 (PMC8476839; doi:10.3389/fmed.2021.723191)
Supplement: Supplementary file 1 [file Data_Sheet_1.pdf]

Supplementary Figure S1. Sensitivity analysis of duration of analgesia

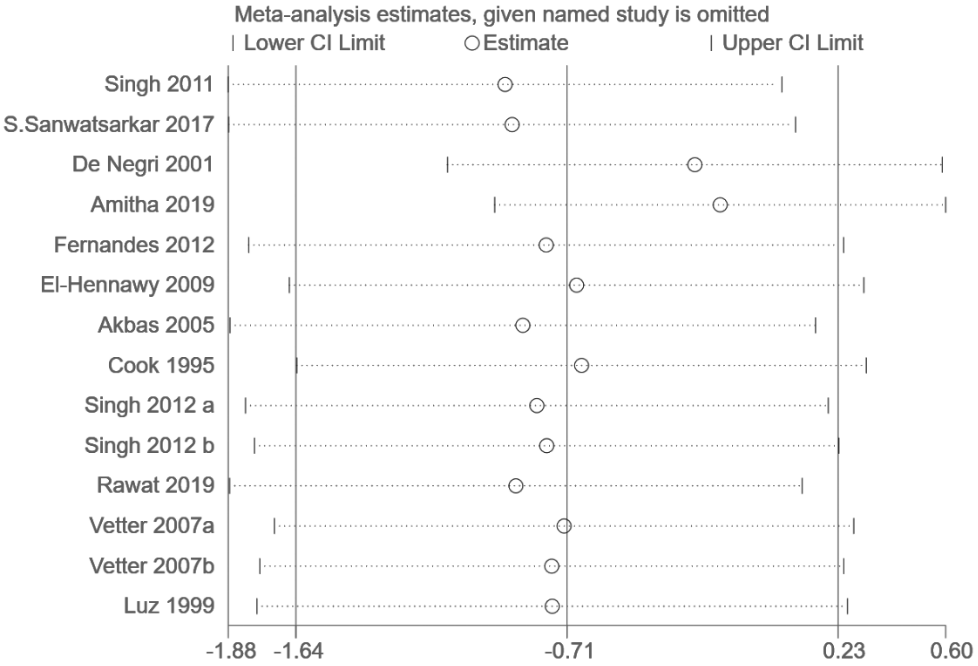

Supplementary Figure S2. Sensitivity analysis of pain score

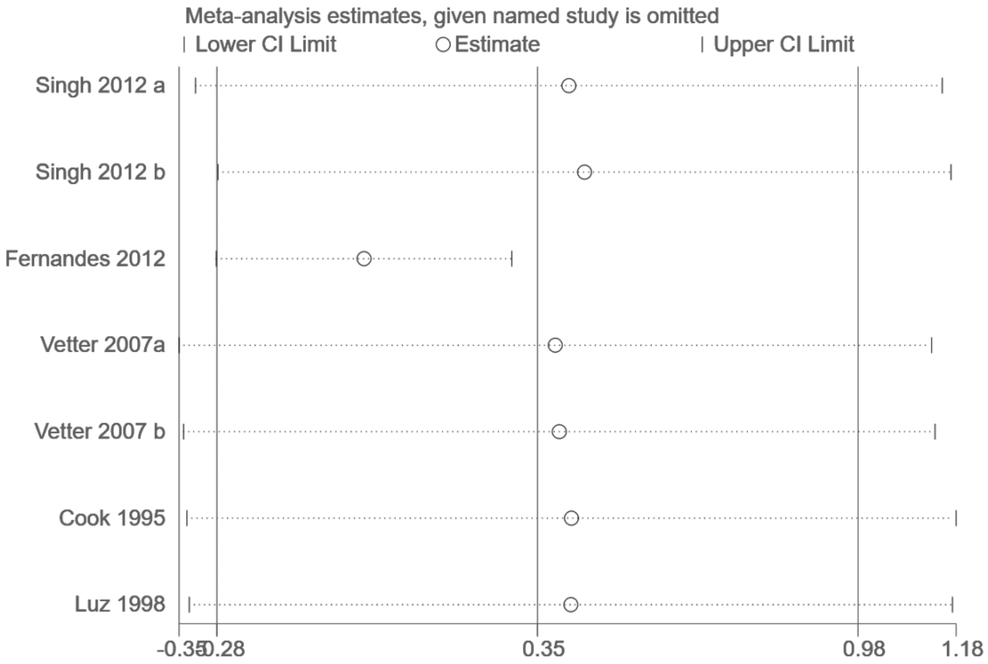

Supplementary Figure S3. Sensitivity analysis of complications

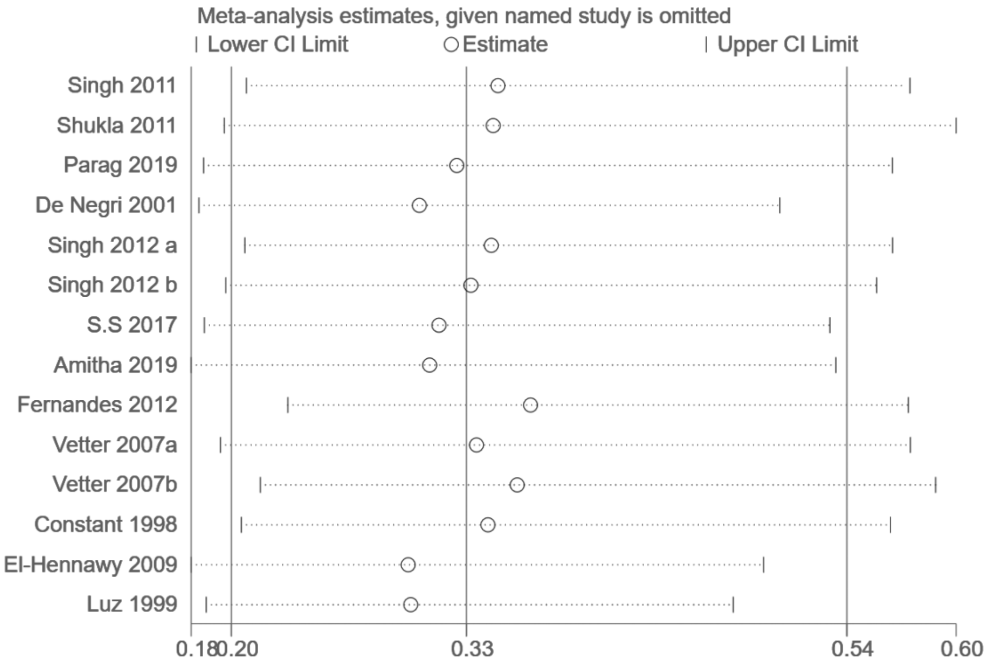

Supplementary Figure S4. Trim and fill of duration of analgesia

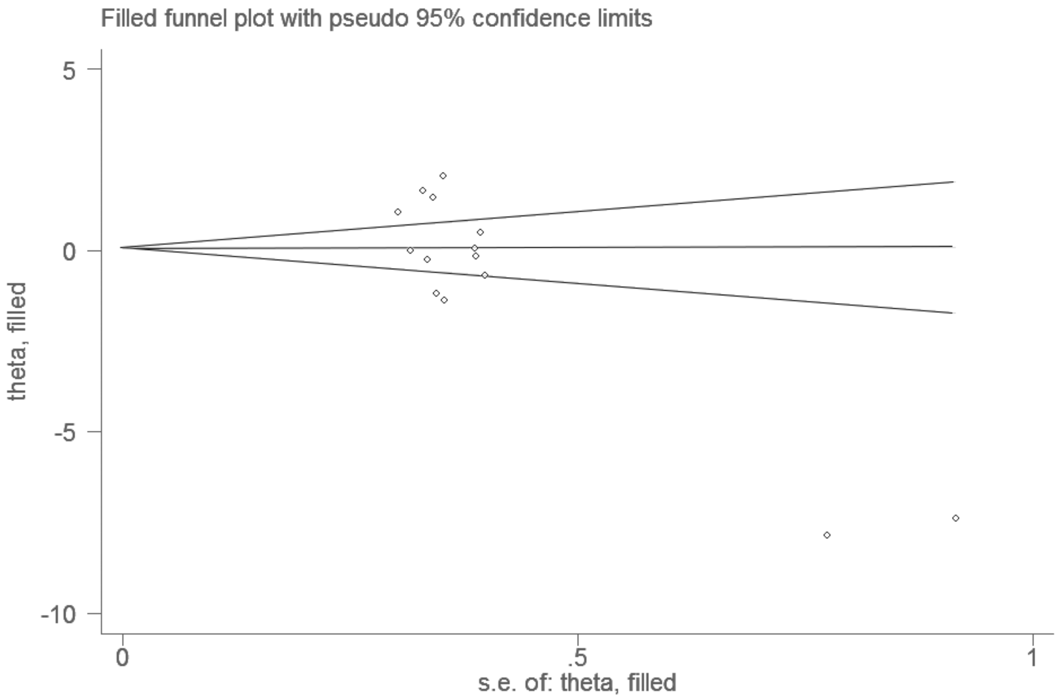

Supplementary Table S1. Rob2 quality assessment for RCTs

Study	Bias arising from the randomization process	Bias due to deviations from intended interventions	Bias due to missing outcome data	Bias in measurement of the outcome	Bias in selection of the reported result	Overall bias
Akbas 2005 (24)	Unclear	Unclear	Low	Low	Low	Unclear
Amitha 2019 (25)	Unclear	Unclear	Low	Low	Low	Unclear
Constant 1998 (29)	Unclear	Low	Low	Low	Low	Low
Cook 1995 (26)	Low	Unclear	Low	Low	Low	Low
De Negri 2001 (30)	Low	Low	Low	Low	Low	Low
El-Hennawy 2009 (19)	Low	Low	Low	Low	Low	Low
Fernandes 2012 (32)	Low	Unclear	Low	Low	Low	Low
Luz 1999 (27)	Unclear	High	Low	Low	Low	High
Parag 2019 (35)	Unclear	Unclear	Low	Low	Low	Unclear
Rawat 2019 (34)	Low	Low	Low	Low	Low	Low
Sanwatsarkar 2017 (9)	Low	Low	Low	Low	Low	Low
Shukla 2011 (38)	Low	Low	Low	Low	Low	Low
Singh 2012 (28)	Unclear	Low	Low	Low	Low	Low
Singh 2011 (33)	Low	Low	Low	Low	Low	Low
Vetter 2007 (6)	Low	Low	Low	Low	Low	Low

Supplementary Table S2. GRADE for each outcome

Title: Clonidine compared to Bupivacaine or Ropivacaine for local anesthetics in caudal block for postoperative analgesia in pediatric surgery

Certainty assessment							№ of patients		Effect		Certainty	Importance
**№ of studies**	**Study design**	**Risk of bias**	**Inconsistency**	**Indirectness**	**Imprecision**	**Other considerations**	**Clonidine**	**Bupivacaine or Ropivacaine**	**Relative (95% CI)**	**Absolute (95% CI)**		
**Pain score (assessed with: FLACC Pain Scale)**												
7	randomised trials	not serious	not serious	serious	not serious	publication bias strongly suspected strong association	98	138	-	SMD **0.354 SD higher** (0.276 lower to 0.983 higher)	⊕⊕⊕◯ MODERATE	CRITICAL
**Duration of analgesia (assessed with: min)**												
14	randomised trials	not serious	not serious	not serious	not serious	publication bias strongly suspected	265	284	-	SMD **0.71 SD lower** (1.644 lower to 0.225 higher)	⊕⊕⊕◯ MODERATE	CRITICAL
**Post-requirement**												
3	randomised trials	not serious	not serious	not serious	not serious	none	45/75 (60.0%)	13/75 (17.3%)	**OR 8.771** (0.696 to 110.577)	**474 more per 1,000** (from 46 fewer to 785 more)	⊕⊕⊕⊕ HIGH	IMPORTANT
**Complications**												
14	randomised trials	not serious	not serious	not serious	not serious	publication bias strongly suspected	62/277 (22.4%)	122/208 (58.7%)	**OR 0.331** (0.205 to 0.536)	**267 fewer per 1,000** (from 361 fewer to 155 fewer)	⊕⊕⊕◯ MODERATE	IMPORTANT

CI: Confidence interval; **SMD:** Standardised mean difference; **OR:** Odds ratio
